# Giving anti-VEGF injections: an update from Burundi

**Published:** 2023-07-07

**Authors:** Jean Claude Niyonzima

**Affiliations:** 1 Consultant Ophthalmologist, VR Surgeon, and Medical Director: Eye and Laser Center and Lecturer: Hope of Africa University, Bujumbura, Burundi.

**Anti-VEGF has become a key tool in DR management.**

Vascular endothelial growth factor (VEGF) is a protein which encourages the growth of new blood vessels. It is produced by many cells in the body, including the retina – usually in response to a lack of oxygen in the surrounding tissues (hypoxia).

If diabetes is not sufficiently controlled, it can lead to hypoxia of the retina and the production of VEGF, which results in the growth of new vessels and diabetic macular oedema, both of which can result in vision loss.

Anti-VEGF drugs are delivered by intravitreal injection, and work by either blocking the production of VEGF in the cell, or by preventing VEGF molecules from binding with blood vessels to trigger the formation of new vessels.

Anti-VEGF injections are becoming increasingly popular as a means of treating DR, because they don’t cause structural changes in the eye (as is the case with laser) or induce cataract (as is the case with steroid treatment).

The first anti-VEGF drug was developed nearly 20 years ago, and more are continually being developed and tested. The three most commonly used anti-VEGF drugs used to treat DR are:

- **Bevacizumab (Avastin).** Widely used “off-label” because of its lower price. There is still no USA Federal Drug Administration (FDA) approval for bevacizumab due to the lack of large, randomised controlled trials. However, health care providers can choose to use it, based on other studies that prove its safety and efficacy.
- **Ranibizumab (Lucentis).** Specifically designed for the treatment of ocular diseases. Approved for intraocular use since 2006.
- **Aflibercept (Eylea).** Also referred to as VEGF Trap-eye; Has FDA approval for diabetic macular oedema treatment.

## Before you start

### History taking

Anti-VEGF intravitreal injections should not be used if there is a history of allergy to the drug or any of its ingredients, if there is infection in the eye or around it, or if there is a severe infection somewhere else in the body.

Anti-VEGF intravitreal injections should be used with caution in pregnant or breastfeeding women, or in patients with a history of heart attack, stroke, uncontrolled angina, or uncontrolled high blood pressure.

### Informed consent

Before patients can consent to receiving anti-VEGF injections, they must be given the correct information. It is helpful to have written information available. Give the patient time to read through it carefully (or have it read to them) and allow time for questions.

Include the following points:

- This procedure is generally safe, although complications may occur.
- Severe (but rare) complications include endophthalmitis, retinal detachment, lens puncture causing cataract, posterior uveitis, bleeding in the eye and increased eye pressure.
- Mild but common complications, which clear a few days after the injection, include discomfort and/or hemorrhage at the site of injection, and seeing floaters.

## On the day

In many countries, intravitreal injections are given in an operating theatre, under a good lighting system or using an operating microscope. However, the procedure can, and has been, carried out successfully in a clinic setting.

### Prepare the patient

Explain to patients what you are going to do, and that they will have to move their eye to a particular position and keep it steady during the procedure.

### Prepare the surgeon and assistants

To minimise the risk of infection, follow the same sterile procedures and use the same personal protective equipment as for eye surgery, including a face mask, hair cover and gloves.

### Prepare the injection

**Figure 1:**
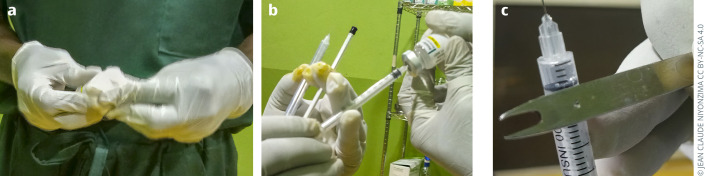
**a.** Disinfecting the vial. **b.** Drawing up the required quantity. **c.** Checking the quantity in the injecting syringe.

Anti-VEGF drugs are available as single and multiple doses. In all forms, the drug should be checked for safety, including the expiry date and vial integrity. Single-dose preparations are ready for use, but multiple-dose vials require additional measures. To use them safely, follow these steps:

1. Keep the vials refrigerated between 2°C and 8°C.
2. Remove the vial just a few minutes before use.
3. Carefully disinfect the vial using cotton wool and 70% isopropyl alcohol (Figure 1a).
4. Draw up the required quantity using a large-bore needle and a 1 ml syringe, then use that syringe to fill as many injecting syringes (30G needle) as needed with individual doses (Figure 1b).
5. Remove any air bubbles in the syringe as this may cause floaters.
6. After the drug is withdrawn, return the vial to the fridge. For convenience, a small fridge can be kept in the operating room. If not, the drug can be kept in a small, insulated box filled with ice packs. Remember to check the temperature at regular intervals.

For bevacizumab, the recommended dose for one eye is 1.25 mg/0.05 ml. It is difficult to draw exactly 0.05 ml, so we usually draw 0.06 ml, represented by the third line on the syringe (Figure 1c).

## The procedure

The procedure requires only **topical anaesthesia**, achieved by instilling a few drops a few minutes before you start.

### Prepare the eye

1. Always confirm the eye to be injected.
2. Clean the eye to be injected using 10% iodine for the peri-ocular surface and 5% iodine eyedrops in the fornix. Alternatively, in patients with iodine hypersensitivity, instill one drop of a topical antibiotic.
3. Most surgeons prefer to drape the eye, although this is not universally done.
4. Use a wire speculum to keep the eyeball accessible and the lashes away from the injection site.

**Figure 2:**
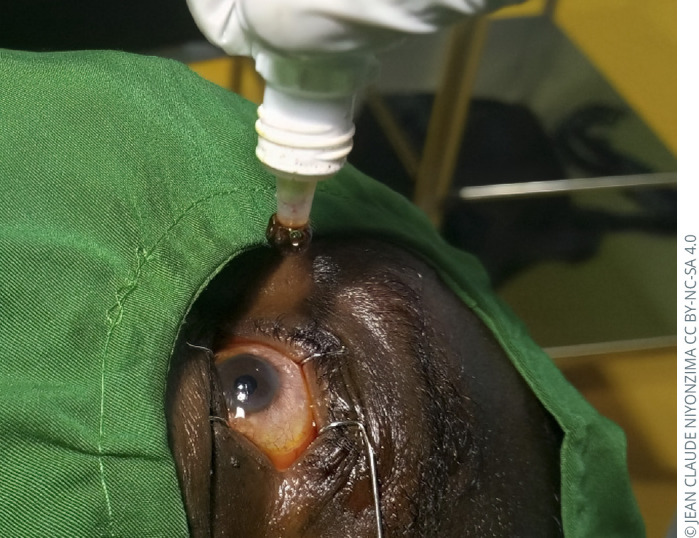
Povidone eye drops instilled on the site of injection and the fornices

**Bilateral injections**

Special caution is needed for patients who require bilateral injections. In normal circumstances, one eye at a time is the safer option. However, in some situations – for example, when patients have difficulties reaching eye care facilities – injecting both eyes can be justified. To minimise the risk of infection, treat each eye using a separate injection set and different batches of the drug.

### Injecting the anti-VEGF

**Figure 3:**
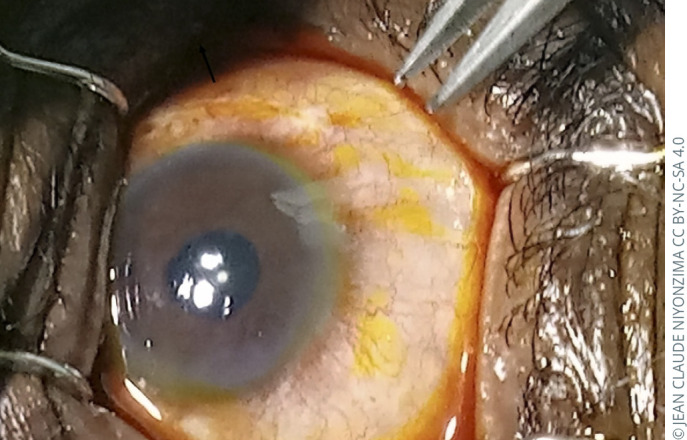
Marking the injection site (arrow) is easy if you use povidone iodine and make a scleral indent.

- Mark the injection site at 3.5–4.0 mm posterior to the limbus in phakic eyes, or at 3.0–3.5 mm posterior to the limbus in aphakic or pseudoaphakic eyes. Marking is made easier by the iodine drop already instilled in the eye.
- Gently position a sterile caliper at the required distance from the limbus and apply a little pressure on the sclera to mark a dot (Figure 3).
- Ask the patient to gaze in an extreme position in order to expose enough sclera.
- Using a cotton tip, slightly push the conjunctiva away over the injection site.
- Insert the 30G needle perpendicularly into the sclera until the tip reaches into the vitreous cavity.
- Slowly inject the drug into the vitreous. You should be able to see small bubbles form behind the crystalline lens.

Withdraw the needle slowly and while applying gentle pressure to the injection site; this helps to minimise reflux and the risk of subconjunctival haemorrhage.

Instil another drop of iodine 5% (or antibiotic, if the patient has iodine hypersensitivity).

## After the procedure

- Check that the patient has visual acuity of hand motion in the treated eye. You can check intraocular pressure (IOP), but be aware that IOP usually goes up for about 30–60 minutes after injection before returning to normal. If injecting 0.05 ml, IOP is rarely a concern, unless it was elevated at the beginning of the procedure.
- Update the patient’s records. These should include the patient’s identity, a summary of clinical information, the date of the procedure, the injected eye, and the batch number of the vial used.

**Figure 4:**
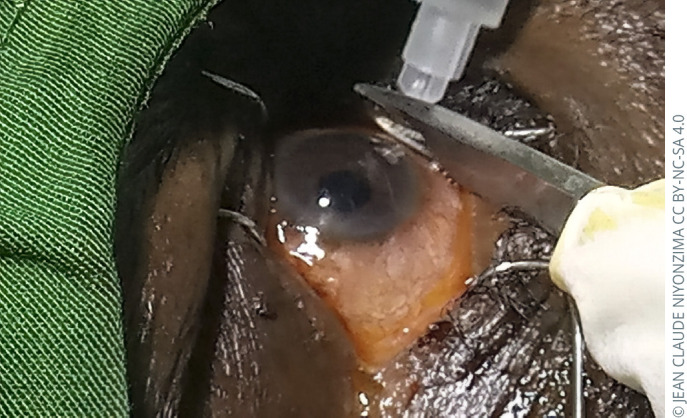
Apply gentle pressure on the site as the needle is being removed.

## At discharge

- Tell the patient to report any increased discomfort or decreased vision as these may suggest a serious complication.
- Clearly communicate to the patient when they must come back and what to expect. These appointments are usually for review and/or for the next injection. It is reasonable to see the patient the same day and give an injection if indicated.

Most patients tend to lose patience after the second or the third injection, so it’s better to be proactive and explain they may need more than six consecutive monthly injections before they get a break. Remind them that diabetic retinopathy is a consequence of a systemic disease that still needs to be taken care of. If you are working in a large hospital, remember to liaise with the diabetologist or physician to make sure the patient’s diabetes is adequately controlled.
